# Supplementary material for: Efficacy of Carbon Nanodots and Manganese Ferrite (MnFe2O4) Nanoparticles in Stimulating Growth and Antioxidant Activity in Drought-Stressed Maize Inbred Lines
Source: Plants (Basel). 2023 Aug 11;12(16):2922. doi: 10.3390/plants12162922 (PMC10458536; doi:10.3390/plants12162922)
Supplement: Supplementary file 1 [file plants-12-02922-s001.zip › plants-2544804-supplementary.pdf]

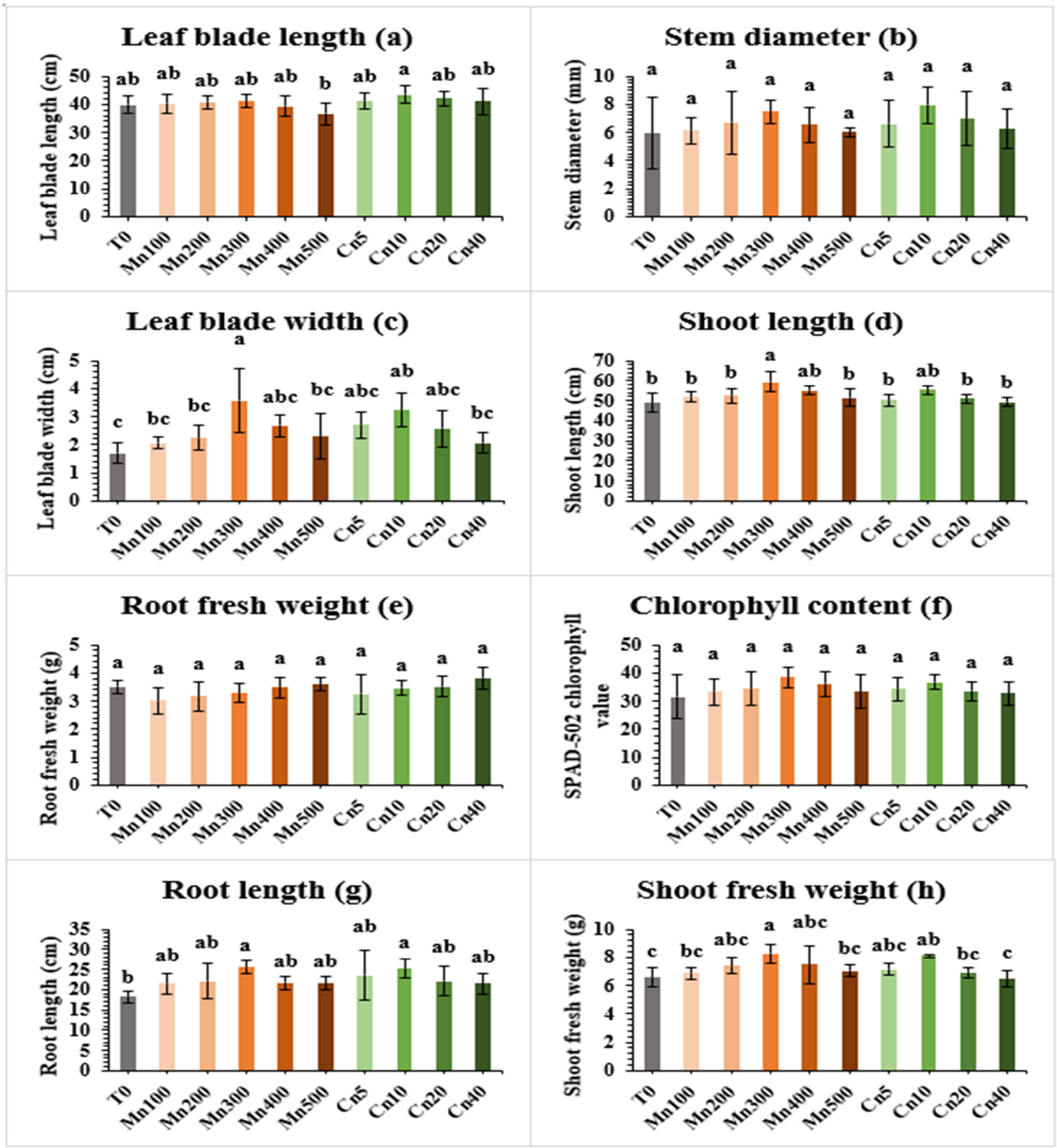

Figure S1. Effects of carbon nanodots (CND) and manganese iron oxide ($MnFe_2O_4$) on leaf blade length (**a**), stem diameter (**b**), leaf blade length (**c**), shoot length (**d**), root fresh weight (**e**), chlorophyll content (**f**), root length (**g**), and shoot length (**h**) of drought stressed HW3 maize inbred line. T0: control treatment; Mn100, Mn200, Mn300, Mn400, and Mn500: foliar application of 100 mg L^{-1} $MnFe_2O_4$ NP, 200 mg L^{-1} $MnFe_2O_4$ NP, 300 mg L^{-1} $MnFe_2O_4$ NP, 400 mg L^{-1} $MnFe_2O_4$ NP and 500 mg L^{-1} $MnFe_2O_4$ NP, respectively; Cn5, Cn10, Cn20, Cn40: foliar application of 5 mg L^{-1}, 10 mg L^{-1}, 20 mg L^{-1}, 40 mg L^{-1} CND, respectively. The results are presented as a means ± standard deviation (n = 3). Different lowercase letters indicate significant differences among different treatments at $p \leq 0.05$.

Table S1. Effects of CND and MnFe2O4 NP on concentrations of different phenolic acids of 41 maize inbred lines under drought stress

**Maize accessions**	**Treatme-nts**	**Phenolic compounds (µg/ml)**					
		**Gallic acid**	**Chlorogenic acid**	**Caffeic acid**	**Syringic acid**	**p-coumaric acid**	**Ferulic acid**
11BS8016-7	$MnFe_2O_4$	2.12 ± 0.02c	1.43 ± 0.03b	1.58 ± 0.02b	0.00 ± 0.00c	1.71 ± 0.02c	0.95 ± 0.01c
	Control	3.36 ± 0.03a	2.21 ± 0.02a	0.00 ± 0.00c	0.45 ± 0.00b	2.80 ± 0.00b	1.62 ± 0.02a
	Cn dots	2.91 ± 0.00b	0.00 ± 0.00c	2.40 ± 0.02a	0.50 ± 0.00a	2.92 ± 0.02a	1.01 ± 0.00b
12BS5076-8	$MnFe_2O_4$	2.32 ± 0.03a	0.00 ± 0.00b	1.78 ± 0.01a	0.45 ± 0.00a	1.17 ± 0.01b	0.74 ± 0.02a
	Control	2.18 ± 0.03c	0.00 ± 0.00b	1.37 ± 0.01c	0.45 ± 0.00b	1.05 ± 0.01c	0.00 ± 0.00b
	Cn dots	2.24 ± 0.01b	5.15 ± 0.03a	1.51 ± 0.01b	0.00 ± 0.00c	1.29 ± 0.02a	0.00 ± 0.00b
12S8052	$MnFe_2O_4$	1.66 ± 0.03c	0.00 ± 0.00c	1.48 ± 0.05c	0.00 ± 0.00c	0.95 ± 0.01c	1.25 ± 0.02b
	Control	2.93 ± 0.02b	1.23 ± 0.01b	2.13 ± 0.02a	0.93 ± 0.02a	1.54 ± 0.02a	1.47 ± 0.01c
	Cn dots	3.48 ± 0.02a	5.18 ± 0.03a	1.64 ± 0.02b	0.46 ± 0.00b	1.33 ± 0.01b	0.84 ± 0.01a
14S8025	$MnFe_2O_4$	1.35 ± 0.00c	0.00 ± 0.00c	1.39 ± 0.02b	0.51 ± 0.00c	0.79 ± 0.01c	0.89 ± 0.04c
	Control	4.67 ± 0.02a	1.41 ± 0.01b	1.16 ± 0.01c	0.77 ± 0.00a	1.52 ± 0.01a	1.46 ± 0.02a
	Cn dots	3.10 ± 0.01b	2.92 ± 0.02a	1.98 ± 0.03a	0.60 ± 0.02b	1.25 ± 0.02b	0.99 ± 0.01b
15RS8039	$MnFe_2O_4$	1.55 ± 0.00c	0.00 ± 0.00c	1.22 ± 0.01c	0.00 ± 0.00c	0.78 ± 0.01c	0.65 ± 0.00b
	Control	2.47 ± 0.01b	1.39 ± 0.03b	1.94 ± 0.02b	0.46 ± 0.00b	0.95 ± 0.00b	1.13 ± 0.04a
	Cn dots	2.77 ± 0.02a	2.65 ± 0.01a	2.02 ± 0.01a	0.60 ± 0.01a	1.13 ± 0.02a	1.10 ± 0.00a
15RS8056	$MnFe_2O_4$	3.00 ± 0.08c	0.00 ± 0.00c	1.11 ± 0.01c	0.00 ± 0.00b	1.55 ± 0.01b	1.33 ± 0.01b
	Control	3.19 ± 0.03b	2.36 ± 0.04b	1.33 ± 0.00b	0.00 ± 0.00b	2.03 ± 0.01a	1.38 ± 0.01a
	Cn dots	3.66 ± 0.02a	4.32 ± 0.03a	2.18 ± 0.01a	0.50 ± 0.00a	1.10 ± 0.01c	1.01 ± 0.01c
15RS8002	$MnFe_2O_4$	2.16 ± 0.00b	0.00 ± 0.00c	0.77 ± 0.01c	0.00 ± 0.00b	1.11 ± 0.01b	1.02 ± 0.00b
	Control	2.35 ± 0.02a	2.17 ± 0.03a	0.96 ± 0.01b	0.00 ± 0.00b	1.38 ± 0.01a	0.00 ± 0.00c
	Cn dots	1.97 ± 0.03c	2.06 ± 0.02b	1.71 ± 0.01a	0.67 ± 0.01a	1.37 ± 0.00a	1.35 ± 0.01a
15S8021-3	$MnFe_2O_4$	1.11 ± 0.01c	0.00 ± 0.00c	1.81 ± 0.03c	0.47 ± 0.01c	0.93 ± 0.01c	0.97 ± 0.02b
	Control	4.25 ± 0.07a	4.42 ± 0.07a	2.79 ± 0.03a	0.90 ± 0.02b	2.13 ± 0.02a	1.51 ± 0.02a
	Cn dots	2.69 ± 0.03b	4.07 ± 0.10b	2.01 ± 0.02b	0.94 ± 0.00a	1.83 ± 0.02b	1.48 ± 0.01a
16CLP23	$MnFe_2O_4$	2.94 ± 0.02c	0.00 ± 0.00c	1.88 ± 0.01b	0.00 ± 0.00c	1.56 ± 0.01b	1.24 ± 0.01b
	Control	4.95 ± 0.02a	1.91 ± 0.03b	2.92 ± 0.03a	0.56 ± 0.00b	1.69 ± 0.01a	1.48 ± 0.02a
	Cn dots	3.39 ± 0.01b	5.53 ± 0.04a	1.55 ± 0.00c	0.59 ± 0.00a	1.36 ± 0.01c	1.23 ± 0.01b
16CLP40	$MnFe_2O_4$	3.29 ± 0.07c	0.00 ± 0.00c	1.11 ± 0.01b	0.00 ± 0.00b	1.60 ± 0.01c	1.24 ± 0.02c
	Control	4.73 ± 0.05a	3.01 ± 0.01b	2.46 ± 0.48a	0.00 ± 0.00b	2.64 ± 0.01a	1.42 ± 0.03a
	Cn dots	3.74 ± 0.01b	4.50 ± 0.00a	2.07 ± 0.01a	0.56 ± 0.01a	2.33 ± 0.00b	1.30 ± 0.00b
17CS5047	$MnFe_2O_4$	3.56 ± 0.03b	0.00 ± 0.00c	1.03 ± 0.02c	0.00 ± 0.00c	1.46 ± 0.00b	1.05 ± 0.02c
	Control	4.02 ± 0.07a	0.87 ± 0.00b	1.47 ± 0.02b	0.57 ± 0.00b	1.86 ± 0.02a	1.28 ± 0.01a
	Cn dots	2.21 ± 0.02c	5.26 ± 0.04a	1.70 ± 0.02a	0.77 ± 0.02a	1.24 ± 0.00c	1.12 ± 0.00b
16S8068-9	$MnFe_2O_4$	2.25 ± 0.00c	0.97 ± 0.01c	1.48 ± 0.01c	0.48 ± 0.00c	0.87 ± 0.00c	0.89 ± 0.01c
	Control	4.82 ± 0.01a	3.17 ± 0.03a	1.60 ± 0.04b	0.56 ± 0.01b	1.76 ± 0.01a	1.23 ± 0.03a
	Cn dots	2.28 ± 0.00b	2.25 ± 0.01b	2.36 ± 0.01a	1.04 ± 0.00a	1.08 ± 0.00b	1.13 ± 0.01b
17CS8006	$MnFe_2O_4$	1.31 ± 0.02c	0.00 ± 0.00c	1.56 ± 0.02a	0.50 ± 0.00b	0.84 ± 0.01c	1.01 ± 0.03a
	Control	5.41 ± 0.04a	3.00 ± 0.05a	0.92 ± 0.02c	0.51 ± 0.00a	1.89 ± 0.01a	0.98 ± 0.02a
	Cn dots	2.74 ± 0.01b	1.73 ± 0.02b	1.13 ± 0.00b	0.00 ± 0.00c	1.85 ± 0.01b	0.88 ± 0.00b
	$MnFe_2O_4$	2.30 ± 0.01c	1.78 ± 0.37a	0.84 ± 0.01b	0.56 ± 0.01b	1.23 ± 0.02c	0.89 ± 0.01b

17CS8 067	Control	2.45 ± 0.07b	1.18 ± 0.00b	0.64 ± 0.01c	0.00 ± 0.00c	1.28 ± 0.00b	0.64 ± 0.00c
	Cn dots	3.25 ± 0.03a	1.90 ± 0.01a	2.13 ± 0.01a	0.59 ± 0.00a	2.01 ± 0.01a	1.41 ± 0.01a
17YS6 032	$MnFe_2O_4$	1.30 ± 0.01c	1.05 ± 0.02c	1.04 ± 0.00b	0.50 ± 0.00a	1.03 ± 0.01c	0.93 ± 0.04b
	Control	3.25 ± 0.04a	3.28 ± 0.03b	0.83 ± 0.01c	0.51 ± 0.00a	1.58 ± 0.01a	1.02 ± 0.02a
	Cn dots	2.71 ± 0.00b	4.13 ± 0.02a	1.89 ± 0.02a	0.31 ± 0.53a	1.29 ± 0.00b	1.06 ± 0.01a
17YS8 003	$MnFe_2O_4$	2.92 ± 0.01b	2.07 ± 0.01a	1.74 ± 0.01a	0.50 ± 0.00a	0.99 ± 0.01b	1.06 ± 0.01a
	Control	3.53 ± 0.02a	1.23 ± 0.02c	1.15 ± 0.01b	0.48 ± 0.01b	1.69 ± 0.02a	1.01 ± 0.01b
	Cn dots	1.02 ± 0.00c	1.46 ± 0.01b	0.62 ± 0.01c	0.00 ± 0.00c	0.73 ± 0.00c	0.00 ± 0.00c
GP3	$MnFe_2O_4$	2.48 ± 0.01b	1.42 ± 0.01c	0.85 ± 0.00c	0.48 ± 0.00c	1.06 ± 0.01c	0.77 ± 0.01c
	Control	2.06 ± 0.02c	4.13 ± 0.04a	1.30 ± 0.03b	0.78 ± 0.01a	1.32 ± 0.01b	1.20 ± 0.01a
	Cn dots	3.92 ± 0.04a	2.04 ± 0.02b	2.14 ± 0.01a	0.58 ± 0.00b	1.58 ± 0.00a	1.08 ± 0.00b
GP5	$MnFe_2O_4$	2.10 ± 0.01c	1.26 ± 0.02c	1.01 ± 0.00c	0.00 ± 0.00c	1.92 ± 0.01c	0.94 ± 0.02b
	Control	3.16 ± 0.01a	3.79 ± 0.05a	1.62 ± 0.03b	0.61 ± 0.01b	2.49 ± 0.00a	1.28 ± 0.03a
	Cn dots	2.97 ± 0.05b	3.03 ± 0.04b	1.90 ± 0.02a	0.96 ± 0.01a	2.03 ± 0.01b	1.26 ± 0.02a
HCW1	$MnFe_2O_4$	2.30 ± 0.02c	1.77 ± 0.02c	0.80 ± 0.00b	0.00 ± 0.00c	1.48 ± 0.01c	0.93 ± 0.02c
	Control	3.95 ± 0.03a	2.63 ± 0.01a	0.69 ± 0.01c	0.48 ± 0.00b	1.65 ± 0.00b	1.11 ± 0.02b
	Cn dots	3.38 ± 0.01b	2.41 ± 0.01b	2.91 ± 0.07a	0.75 ± 0.00a	1.67 ± 0.00a	1.46 ± 0.01a
HCW2	$MnFe_2O_4$	2.31 ± 0.06c	2.89 ± 0.02a	1.13 ± 0.01b	0.00 ± 0.00c	1.30 ± 0.01c	1.16 ± 0.01c
	Control	5.01 ± 0.08a	1.40 ± 0.02b	1.16 ± 0.03b	0.52 ± 0.00b	2.33 ± 0.01a	1.83 ± 0.03a
	Cn dots	3.48 ± 0.04b	0.00 ± 0.00c	3.01 ± 0.48a	0.59 ± 0.00a	1.57 ± 0.00b	1.52 ± 0.02b
HCW3	$MnFe_2O_4$	2.21 ± 0.00c	1.18 ± 0.02c	0.64 ± 0.00c	0.51 ± 0.00c	1.18 ± 0.01c	1.13 ± 0.01c
	Control	3.67 ± 0.02a	4.50 ± 0.10a	1.10 ± 0.01b	0.66 ± 0.01a	2.26 ± 0.02b	1.39 ± 0.02b
	Cn dots	3.58 ± 0.01b	2.22 ± 0.02b	2.07 ± 0.02a	0.57 ± 0.01b	2.35 ± 0.02a	1.64 ± 0.03a
HCW4	$MnFe_2O_4$	0.97 ± 0.01c	0.00 ± 0.00c	0.00 ± 0.00c	0.64 ± 0.00a	0.91 ± 0.00c	0.70 ± 0.01c
	Control	4.84 ± 0.03a	1.94 ± 0.01b	0.77 ± 0.01b	0.46 ± 0.00b	1.93 ± 0.01a	0.74 ± 0.00b
	Cn dots	2.10 ± 0.02b	4.57 ± 0.06a	1.87 ± 0.03a	0.00 ± 0.00c	1.32 ± 0.01b	0.89 ± 0.00a
HCW5	$MnFe_2O_4$	2.08 ± 0.02c	1.02 ± 0.02c	0.76 ± 0.02a	0.48 ± 0.01c	1.11 ± 0.01c	0.96 ± 0.02c
	Control	3.56 ± 0.02a	3.03 ± 0.08b	1.02 ± 0.03a	0.54 ± 0.00b	1.69 ± 0.01a	1.14 ± 0.00b
	Cn dots	2.58 ± 0.02b	6.10 ± 0.02a	1.49 ± 1.29a	0.81 ± 0.01a	1.64 ± 0.01b	1.46 ± 0.01a
HF12	$MnFe_2O_4$	2.31 ± 0.05b	0.95 ± 0.01b	0.77 ± 0.01b	0.55 ± 0.01b	1.36 ± 0.01b	1.13 ± 0.01a
	Control	3.55 ± 0.02a	0.86 ± 0.02b	0.68 ± 0.01c	0.51 ± 0.01c	1.92 ± 0.02a	0.75 ± 0.00c
	Cn dots	2.25 ± 0.03b	6.34 ± 0.11a	1.88 ± 0.02a	0.66 ± 0.01a	1.37 ± 0.01b	1.03 ± 0.01b
HF22	$MnFe_2O_4$	2.11 ± 0.02c	3.13 ± 0.01a	0.73 ± 0.01c	0.55 ± 0.00b	1.24 ± 0.00c	1.12 ± 0.03b
	Control	3.11 ± 0.02b	1.57 ± 0.02c	0.92 ± 0.01b	0.52 ± 0.00c	2.32 ± 0.01a	1.11 ± 0.02b
	Cn dots	3.47 ± 0.02a	2.54 ± 0.01b	2.62 ± 0.02a	0.87 ± 0.01a	1.87 ± 0.00b	1.33 ± 0.00a
HW1	$MnFe_2O_4$	3.03 ± 0.03c	1.78 ± 0.03b	1.20 ± 0.04b	0.00 ± 0.00c	1.63 ± 0.03b	1.24 ± 0.01b
	Control	5.10 ± 0.07a	1.54 ± 0.01c	0.74 ± 0.00c	0.48 ± 0.00a	2.46 ± 0.04a	1.39 ± 0.01a
	Cn dots	3.38 ± 0.03b	2.01 ± 0.01a	1.70 ± 0.03a	0.45 ± 0.00b	1.28 ± 0.00c	1.08 ± 0.01c
HW10	$MnFe_2O_4$	2.51 ± 0.04b	0.00 ± 0.00b	1.00 ± 0.01c	0.51 ± 0.00b	1.19 ± 0.01c	1.18 ± 0.01c
	Control	3.94 ± 0.03a	0.00 ± 0.00b	1.09 ± 0.01b	0.54 ± 0.01a	3.14 ± 0.03a	1.43 ± 0.01a
	Cn dots	2.41 ± 0.02c	2.58 ± 0.03a	2.06 ± 0.00a	0.50 ± 0.00b	1.38 ± 0.00b	1.27 ± 0.02b
HW11	$MnFe_2O_4$	1.90 ± 0.03c	1.03 ± 0.04b	1.14 ± 0.01c	0.50 ± 0.00a	1.35 ± 0.01c	1.11 ± 0.01a
	Control	3.70 ± 0.02a	0.99 ± 0.01b	1.53 ± 0.03b	0.00 ± 0.00b	2.92 ± 0.01a	0.70 ± 0.01c
	Cn dots	3.63 ± 0.04b	2.02 ± 0.03a	2.19 ± 0.01a	0.00 ± 0.00b	1.96 ± 0.00b	1.05 ± 0.00b
HW12	$MnFe_2O_4$	1.99 ± 0.01c	1.44 ± 0.03c	0.64 ± 0.00c	0.50 ± 0.00a	0.87 ± 0.01b	1.25 ± 0.02a

	Control	4.24 ± 0.05a	2.22 ± 0.06a	1.01 ± 0.01b	0.49 ± 0.00b	2.14 ± 0.02a	1.07 ± 0.05c
	Cn dots	3.90 ± 0.03b	2.08 ± 0.01b	1.39 ± 0.00a	0.00 ± 0.00c	2.13 ± 0.02a	1.15 ± 0.00b
HW15	$MnFe_2O_4$	1.00 ± 0.01c	0.00 ± 0.00b	1.40 ± 0.06b	0.52 ± 0.00a	1.00 ± 0.03c	0.00 ± 0.00b
	Control	3.78 ± 0.03a	0.00 ± 0.00b	0.76 ± 0.00c	0.49 ± 0.00b	1.53 ± 0.00a	0.78 ± 0.03a
	Cn dots	2.94 ± 0.05b	2.77 ± 0.01a	2.17 ± 0.09a	0.49 ± 0.01b	1.28 ± 0.00b	0.84 ± 0.08a
HW16	$MnFe_2O_4$	1.92 ± 0.04c	1.08 ± 0.08c	1.96 ± 0.05a	0.71 ± 0.03a	1.08 ± 0.01c	0.72 ± 0.01c
	Control	2.88 ± 0.03a	1.67 ± 0.03b	0.96 ± 0.04c	0.00 ± 0.00b	1.53 ± 0.01b	0.89 ± 0.03b
	Cn dots	2.00 ± 0.02b	2.41 ± 0.06a	1.47 ± 0.00b	0.00 ± 0.00b	1.67 ± 0.01a	0.94 ± 0.01a
HW17	$MnFe_2O_4$	2.55 ± 0.01c	0.97 ± 0.02c	2.02 ± 0.09a	0.45 ± 0.00c	1.35 ± 0.02c	1.18 ± 0.02b
	Control	3.09 ± 0.07b	1.43 ± 0.02b	1.62 ± 0.01c	0.52 ± 0.02a	1.45 ± 0.01b	1.16 ± 0.02b
	Cn dots	3.53 ± 0.03a	5.53 ± 0.07a	1.82 ± 0.00b	0.49 ± 0.00b	2.01 ± 0.01a	1.24 ± 0.00a
HW18	$MnFe_2O_4$	2.33 ± 0.00b	0.92 ± 0.02c	0.70 ± 0.00c	0.51 ± 0.01a	0.98 ± 0.01c	0.93 ± 0.01a
	Control	2.46 ± 0.03a	2.80 ± 0.06b	1.05 ± 0.01b	0.00 ± 0.00b	1.11 ± 0.01b	0.00 ± 0.00c
	Cn dots	1.87 ± 0.01c	4.13 ± 0.03a	1.17 ± 0.01a	0.00 ± 0.00b	1.27 ± 0.01a	0.77 ± 0.01b
HW19	$MnFe_2O_4$	2.41 ± 0.02b	1.21 ± 0.01c	1.17 ± 0.01b	0.53 ± 0.00a	1.33 ± 0.01b	1.20 ± 0.01a
	Control	2.78 ± 0.03a	5.13 ± 0.03a	1.18 ± 0.00a	0.49 ± 0.01b	1.40 ± 0.02a	1.02 ± 0.00b
	Cn dots	1.86 ± 0.05c	4.02 ± 0.01b	1.19 ± 0.01a	0.45 ± 0.00c	1.13 ± 0.00c	0.80 ± 0.00c
HW3	$MnFe_2O_4$	1.84 ± 0.01c	1.12 ± 0.03b	0.95 ± 0.02c	0.49 ± 0.00b	1.05 ± 0.00c	1.14 ± 0.02a
	Control	3.73 ± 0.06b	1.24 ± 1.08b	1.65 ± 0.01a	0.53 ± 0.00a	2.40 ± 0.04b	1.07 ± 0.01b
	Cn dots	4.38 ± 0.01a	4.57 ± 0.05a	1.38 ± 0.03b	0.48 ± 0.00c	2.52 ± 0.02a	0.80 ± 0.00c
HW4	$MnFe_2O_4$	1.68 ± 0.01c	0.00 ± 0.00c	0.00 ± 0.00c	0.66 ± 0.01b	1.13 ± 0.01c	1.09 ± 0.01c
	Control	3.33 ± 0.06b	2.28 ± 0.09b	1.92 ± 0.03a	0.69 ± 0.01a	1.64 ± 0.01b	1.24 ± 0.00b
	Cn dots	3.54 ± 0.02a	3.85 ± 0.05a	1.32 ± 0.00b	0.47 ± 0.00c	2.45 ± 0.01a	1.69 ± 0.01a
HW7	$MnFe_2O_4$	1.99 ± 0.01c	0.00 ± 0.00b	0.00 ± 0.00c	0.72 ± 0.00a	1.12 ± 0.01b	0.86 ± 0.00c
	Control	4.18 ± 0.02a	0.00 ± 0.00b	1.87 ± 0.02b	0.48 ± 0.00b	1.83 ± 0.01a	1.80 ± 0.01a
	Cn dots	3.71 ± 0.01b	8.71 ± 0.02a	2.68 ± 0.02a	0.00 ± 0.00c	0.87 ± 0.00c	1.48 ± 0.00b
HW8	$MnFe_2O_4$	2.21 ± 0.01c	1.09 ± 0.03c	1.25 ± 0.02b	0.48 ± 0.00a	0.91 ± 0.02c	1.27 ± 0.01a
	Control	3.34 ± 0.09b	1.55 ± 0.00b	1.00 ± 0.01c	0.00 ± 0.00b	1.79 ± 0.01b	0.80 ± 0.00c
	Cn dots	4.67 ± 0.06a	6.75 ± 0.04a	2.60 ± 0.02a	0.00 ± 0.00b	1.94 ± 0.01a	1.23 ± 0.01b
HW9	$MnFe_2O_4$	0.93 ± 0.01c	1.20 ± 0.01c	1.22 ± 0.02a	0.52 ± 0.00a	0.88 ± 0.01c	0.80 ± 0.01b
	Control	2.38 ± 0.05b	1.77 ± 0.03b	1.17 ± 0.00b	0.47 ± 0.00a	1.14 ± 0.00b	0.00 ± 0.00c
	Cn dots	3.09 ± 0.02a	5.47 ± 0.04a	1.13 ± 0.01c	0.33 ± 0.29a	1.85 ± 0.03a	1.32 ± 0.01a
KL103	$MnFe_2O_4$	0.90 ± 0.01c	1.00 ± 0.02b	0.60 ± 0.00b	0.52 ± 0.02a	0.84 ± 0.01c	0.81 ± 0.00b
	Control	4.65 ± 0.06a	0.00 ± 0.00c	1.38 ± 0.01a	0.00 ± 0.00b	3.69 ± 0.01a	0.73 ± 0.00c
	Cn dots	3.30 ± 0.02b	3.46 ± 0.04a	0.00 ± 0.00c	0.00 ± 0.00b	2.06 ± 0.00b	1.05 ± 0.01a
KW7	$MnFe_2O_4$	3.83 ± 0.01b	0.00 ± 0.00c	0.81 ± 0.00b	0.00 ± 0.00b	2.17 ± 0.01b	0.72 ± 0.00a
	Control	4.52 ± 0.01a	1.20 ± 0.01a	1.27 ± 0.02a	0.00 ± 0.00b	4.06 ± 0.06a	0.80 ± 0.10a
	Cn dots	3.61 ± 0.04c	0.97 ± 0.01b	0.62 ± 0.00c	0.46 ± 0.00a	1.47 ± 0.01c	0.00 ± 0.00b

Results are presented as the means ± standard deviation ($n = 3$). Different lowercase letters within each phenolic acid result indicate statistically significant differences among applied treatments on each line at $p \leq 0.05$.

Table S2. Maize inbred lines and F1 hybrids developed in the Maize Experimental Station

Entry No.	Inbred line	Parent for F1 varieties
1	11BS8016-7	Gangwonchal 43 ♀
2	12BS5076-8	Gangwonchal 43 ♂
3	12S8052	Gangwonchal 34 ♂
4	14S8025	Gangwonchal 58 ♂
5	15RS8039	Gangwonchal 51 ♀
6	15RS8056	Gangwonchal 51 ♂
7	15RS8002	Gangwonchal 57 ♀
8	15S8021-3	Gangwonchal 48 ♀
9	16CLP23	Saekchalgyo 109 ♀
10	16CLP40	Saekchalgyo 105 ♀
11	17CS5047	Saekchalgyo 105 ♂
12	16S8068-9	Gangwonchal 48 ♂
13	17CS8006	Saekchalgyo 109 ♂
14	17CS8067	Saekchalgyo 56 ♀
15	17YS6032	Gangwonchal 60 ♀
16	17YS8003	Gangwonchal 60 ♂
17	GP3	Oryun2ho ♂
18	GP5	Oryun2ho ♀
19	HCW1	Cheongchunchal ♀
20	HCW2	Cheongchunchal ♂
21	HCW3	Hongmichal ♀
22	HCW4	Hongmichal ♂
23	HCW5	Mihongchal ♂
24	HF12	Dreamok ♂
25	HF22	Dreamok ♀
26	HW1	Dumechal ♀
27	HW10	Heugjeom2ho ♀
28	HW11	Arichal ♂
29	HW12	Gangwonchal 34 ♀, Arichal ♀, Jangsuchal ♀
30	HW15	Jangsuchal ♂
31	HW16	Goldchal ♀
32	HW17	Goldchal ♂
33	HW18	Gangwonchal 57 ♂, Mihyeonchal ♀
34	HW19	Mihyeonchal ♂, Bunongchal ♂
35	HW3	Mibaek2ho ♂, Mibaekchal ♀, Saekchalgyo 56 ♂
36	HW4	Mibaekchal ♂
37	HW7	Miheugchal ♀, Heugjeom2ho ♂
38	HW8	Miheugchal ♂
39	HW9	Gangwonchal 46 ♂, Mibaek2ho ♀, Mihongchal ♀, Bunongchal ♀
40	KL103	Heugjeomchal ♀
41	KW7	Dumechal ♂, Heugjeomchal ♂
